# Effects of Two Manure Additives on Methane Emissions from Dairy Manure

**DOI:** 10.3390/ani10050807

**Published:** 2020-05-07

**Authors:** Jessie Cluett, Andrew C. VanderZaag, Hambaliou Baldé, Sean McGinn, Earl Jenson, Alexander C. Hayes, Sylvanus Ekwe

**Affiliations:** 1 Department of Physics and Atmospheric Science, Dalhousie University, Halifax, NS B3H 4R2, Canada; jessie.cluett@dal.ca; 2 Ottawa Research and Development Centre, Agriculture and Agri-Food Canada, Ottawa, ON K1A 0C6, Canada; hambaliou.balde@canada.ca; 3 Lethbridge Research and Development Centre, Agriculture and Agri-Food Canada, Lethbridge, AB T1J 4T1, Canada; sean.mcginn@canada.ca; 4 InnoTech Alberta, Vegreville, AB T9C 1T4, Canada; earl.jenson@innotechalberta.ca (E.J.); alexander.hayes@innotechalberta.ca (A.C.H.); sylvanus.ekwe@innotechalberta.ca (S.E.)

**Keywords:** dairy manure, manure additives, liquid manure storage, methane emissions, Biochemical Methane Potential (BMP)

## Abstract

**Simple Summary:**

Livestock farms often store liquid manure until it can be used to fertilize crops. During anaerobic storage, the manure produces methane, which is a greenhouse gas. Many livestock farms add special chemical products to the manure that are said to control odours or increase fertilizer value. We wanted to know if these additives change the amount of methane produced. Two additives that are commonly used by farmers in many countries were tested in the laboratory. We mixed liquid dairy manure with different amounts of these products and measured the amount of methane produced over 30 to 90 days. Results were then compared to the same manure without any product. These tests were done at two temperatures, around 37 °C (a typical biodigester temperature), and 20 °C (a typical manure storage temperature). We also compared the chemical and physical properties of manure. We found that adding these products did not change the amount of methane produced, and it did not change the chemical and physical properties of the manure related to methane production. These findings suggest that farms using these products can be expected to have normal methane emissions from stored manure.

**Abstract:**

Liquid manure is a significant source of methane (CH_4_), a greenhouse gas. Many livestock farms use manure additives for practical and agronomic purposes, but the effect on CH_4_ emissions is unknown. To address this gap, two lab studies were conducted, evaluating the CH_4_ produced from liquid dairy manure with Penergetic-g^®^ (12 mg/L, 42 mg/L, and 420 mg/L) or AgrimestMix^®^ (30.3 mL/L). In the first study, cellulose produced 378 mL CH_4_/g volatile solids (VS) at 38 °C and there was no significant difference with Penergetic-g^®^ at 12 mg/L or 42 mg/L. At the same temperature, dairy manure produced 254 mL CH_4_/g VS and was not significantly different from 42 mg/L Penergetic-g^®^. In the second lab study, the dairy manure control produced 187 mL CH_4_/g VS at 37 °C and 164 mL CH_4_/g VS at 20 °C, and there was no significant difference with AgrimestMix (30.3 mL/L) or Penergetic-g^®^ (420 mg/L) at either temperature. Comparisons of manure composition before and after incubation indicated that the additives had no effect on pH or VS, and small and inconsistent effects on other constituents. Overall, neither additive affected CH_4_ production in the lab. The results suggest that farms using these additives are likely to have normal CH_4_ emissions from stored manure.

## 1. Introduction

Methane (CH_4_) emissions from manure management are an important contributor to the greenhouse gas budget for dairy farms, and the agricultural sector [1,2]. Since CH_4_ has a global warming potential 34 times greater than CO_2_ (100-year time horizon, including climate–carbon feedback), it is important to characterize emissions from manure management and identify mitigation strategies [3,4]. To this end, our research team at Agriculture and Agri-Food Canada has established a network of research sites to measure CH_4_ emissions from dairy and swine farms in multiple regions across Canada. The goal is to better understand the CH_4_ emissions from liquid manure, and the response to regional climate and management.

While establishing this research network, we learned that many livestock operations in our network were currently using manure additives (i.e., three farms out of seven). The farm operators were using these products to reduce odours, reduce the need for agitation (increased manure homogeneity and less crusting), and to increase nutrient availability for crop growth. There are many different types of additives available such as vermiculite, slag waste, biomass-derived carbon, and transition metal compounds, each with their own benefits and effects on the manure slurry during anaerobic storage. Other methods of treating manure, such as anaerobic digestion to produce electricity or natural gas (biogas) have limited uptake on Canadian farms due to the high capital costs, among other things. Thus, this study is focused on additives used during manure storage. Although the manufacturers of these products do not make claims related to CH_4_ emissions, we were interested to determine whether these additives could impact emissions from liquid manure. Therefore, a series of laboratory experiments were conducted to help understand whether additives affect CH_4_ emissions. Results from the lab studies will also help inform our on-farm studies. Additives such as those mentioned above may be produced or available in many countries around the world, therefore, this study has implications for any countries in which the additives are sold.

There have been some studies on the effects of several manure additives on emissions. McCrory and Hobbs summarized the effects of adding acidifying or absorbent materials to reduce ammonia (NH_3_) emissions [5], while others have observed acidification led to reduced CH_4_ emissions and inhibition of methanogens [6]. A study by Matulaitis et al. [7] tested microbial-based additives, finding that they did not result in a significant difference in NH_3_, CH_4_, H_2_S, CO_2_, CO, or NO emissions for untreated swine or dairy manure stored in buckets at 5 °C, 15 °C, and 25 °C for 29 days. Recently, commercial additives EU200^®^, Bio-buster^®^, and JASS^®^ were studied for their effects on liquid manure constituent concentrations and gaseous emissions (CH_4_, CO_2_, N_2_O, NH_3_) [8]. The authors concluded that the additives had no effect on constituents or emissions during storage. Other studies found that some additives are used as accelerants and were found to increase total biogas production. Some examples of studied accelerants include vermiculite with a 26–51% increase, a bio-based carbon additive reported a 77% increase, and various nano-scale transition metal carbides were found to increase biogas production by 57.5–70%. [9,10,11]. When adding steel slag to an anaerobic digester, there was an 83.5–134% increase in total biogas production [12].

In our study, two specific products were selected because they were most commonly used at our cooperating farms: AgrimestMix^®^ (Rinagro B.V., Piaam, Netherlands) and Penergetic-g^®^ (Penergetic International, Romanshorn, Switzerland). Considering the widespread commercial use of these additives, little research has been done to test their effects on manure characteristics and emissions processes. Some manufacturer-sponsored studies have been done, however, these only concerned ammonia emissions. Buro Blauw B.V. [13] reported a 26% reduction in ammonia emissions with AgrimestMix^®^ added to the slurry, while Van der Stelt et al. [14] found no significant decrease in NH_3_ emissions and no change in crusting when using AgrimestMix^®^ combined with Effective Micro-organisms^®^. No studies on the effect of Penergetic-g^®^ on manure characteristics or gas emissions were found in the Web of Science or Scopus databases or on the manufacturer’s website.

Therefore, the objective of this study was to determine the effect of two commonly used manure additives, Penergetic-g^®^ and AgrimestMix^®^, on CH_4_ emissions from liquid dairy manure. The hypothesis was that these additives would reduce CH_4_ emissions.

## 2. Materials and Methods 

Two laboratory studies were conducted. Study #1 took place in 2017 at the InnoTech Alberta laboratory in Vegreville, Alberta, Canada. Study #2 took place in 2019 at the Agriculture and Agri-Food Canada laboratory in Ottawa, Ontario, Canada. In each study, manure from a local dairy farm was used, as described below. At the time of Study #1, Penergetic-g^®^ was the only additive used on farms in our research network, therefore it was the focus of that study. At the time of Study #2, one farm in our network had begun using AgrimestMix^®^, therefore this additive was also studied. The doses of Penergetic-g^®^ in Study #1 were selected to bracket the recommended range of initial application rates, while a much higher dose was used in Study #2 since no effect had been seen in Study #1.

### 2.1. Farm Descriptions

Study #1: liquid dairy manure was obtained from a naturally ventilated free-stall dairy farm near Leduc, Alberta. The farm had 82 lactating Holstein–Friesian cows, 55 dry cows, and 32 heifers on-site. The total mixed ration contained barley silage, dry hay, and concentrate. The dry matter intake by milking cows was 25 kg/d/cow. Milk production averaged 37 L/d/cow. Wood shavings were used as bedding. Manure was scraped from the alleys to an under-barn concrete pit where it was stored for less than a week as it flowed by gravity to an outdoor earthen manure storage. A composite sample of manure slurry was collected from 10 locations in the under-barn concrete pit in the winter of 2017, mixed in a 20 L bucket, and then stored at 4 °C. This sample was then subdivided and frozen for 3 weeks until the start of the study.

Study #2: liquid dairy manure was obtained from a naturally ventilated free-stall dairy farm near Ottawa, Ontario. The farm had 172 lactating Holstein–Friesian cows, 8 dry cows, and 80 heifers on-site as described in the 2016 study by Baldé et al. [15]. The total mixed ration contained Alfalfa/grass hay, corn silage, and high moisture corn. In addition, concentrate pellets were fed at the milking robots. In total, the dry matter intake was 24 kg/d/milking cow. Milk production averaged 35 L/d/cow. Wood shavings were used as bedding. Manure was scraped from the alleys every 15-min and pumped directly to a manure tank. The manure used in this study was sampled from the storage tank in the spring of 2019 and stored at 4 °C fridge until the start of the study. 

### 2.2. Manure Additives

A jug of AgrimestMix^®^ was obtained from the Canadian distributor (Turin, AB, Canada). According to the manufacturer, the product is composed of an activated natural mineral blend. It is said to stimulate the growth of micro-organisms that transform organic bound nitrogen into ammonium nitrogen. It is stated that the product will prevent crusting, reduce odour and ammonia emissions from manure storage tanks as well as increase the quality of the manure as a fertilizer [16]. The manufacturer recommends an initial application rate of 30.3 mL of AgrimestMix^®^ per liter of manure, followed by a weekly maintenance dose of 6 mL/L of manure. In this study, we used the initial application rate of 30.3 mL/L. As no new manure was added during the study, no weekly maintenance dose was added.

A box of Penergetic-g^®^ was obtained from the Ontario distributor. According to the manufacturer, the product acts as a catalyst to create aerobic conditions that favors the growth of beneficial microbes [17]. Penergetic Canada states that the product will optimize liquid manure by homogenizing the slurry, mitigating the formation of a floating crust, and optimizes manure nutrients by preventing the loss of ammonium nitrogen from slurry. The composition of Penergetic-g^®^ is proprietary, so the compounds used are not known. The manufacturer recommends an initial dosing rate of 15 to 20 mg of Penergetic-g^®^ per liter of manure and a weekly maintenance dosage of 5 g per dairy cow [17]. In this study, a range of dosages were tested from 12 to 420 mg/L. As no new manure was added during the study, no weekly maintenance dose was added after the initial dose. The range of application rates in product literature was between 15 to 20 mg/L, however, following discussion with sales representatives it was indicated that sometimes a double dose (40 mg/L) is recommended at start-up. The rates used in the study were 12 and 42 mg/L which bracket the recommended rates. After finding no effect in the first study, we increased the highest rate by 10× to 420 mg/L to see if an effect could be observed.

### 2.3. Methane Emissions Laboratory Setup

#### 2.3.1. Study #1

**Figure 1:**
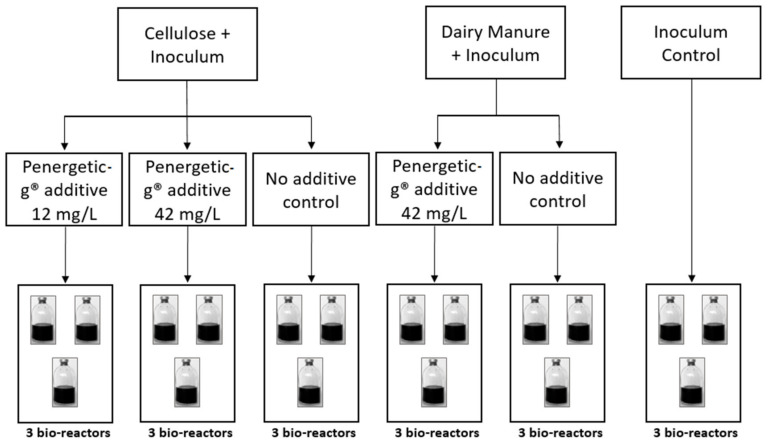
Biochemical methane potential (BMP) trial set-up with 18 bioreactors in InnoTech Alberta’s batch culture set-up. Each additive treatment, the positive control (no additive), and the inoculum control were done in triplicate

A biochemical methane potential (BMP) batch test was used to investigate the effect of the Penergetic-g^®^ additive on CH_4_ emissions from two different substrates, dairy manure from the Alberta Farm, and a research grade microcrystalline cellulose with a known potential to produce CH_4_ (Alfa Aesar, Fisher Scientific, Ottawa, ON, Canada). The test was performed using 18 low-pressure bioreactors in InnoTech Alberta’s BMP batch culture system (Figure 1). Each 2 L bioreactor was filled with 1 L culture volume, comprising a mixture of substrate, methanogenic inoculum (to speed up the establishment of a microbial community for methanogenesis) and water with a substrate/inoculum volatile solids (VS) ratio of 0.6, and 1 L of headspace. Penergetic-g^®^ was added to the cellulose cultures at two rates, 12 mg/L and 42 mg/L. For the cultures with manure as substrate, only the higher dosage of 42 mg/L was evaluated. Controls without additive were included for both cellulose and manure substrates. Inoculum controls were included so the CH_4_ contribution from inoculum could be subtracted from each reactor. Methanogenic inoculum was incubated and allowed to degas at room temperature for two days prior to use. All cultures were prepared in triplicate. The bioreactors were incubated for 26 days for cellulose, and 33 days for dairy manure, at a mesophilic temperature of 38 °C (Figure 1). The cellulose and manure methane trials were stopped when daily gas production fell below 1% of the total cumulative gas produced.

#### 2.3.2. Study #2

**Figure 2:**
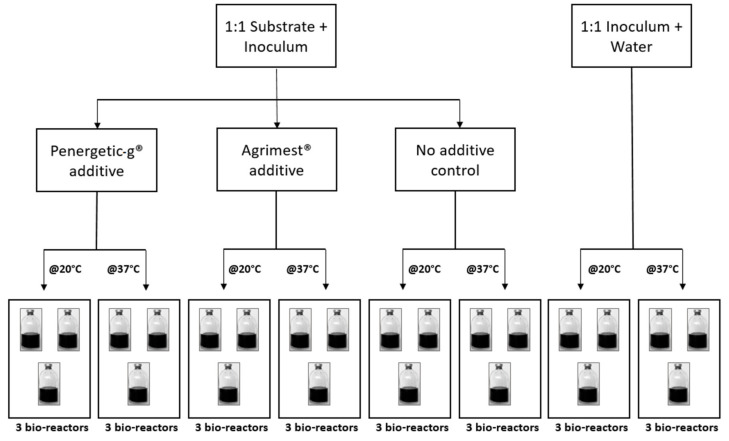
Biochemical methane potential (BMP) trial set-up with 24 bottles divided between two incubation temperatures, 20 °C and 37 °C. The substrate was liquid dairy manure. Each additive treatment, the manure control (no additive), and the inoculum control were done in triplicate

The methane potential was determined for liquid dairy manure with two manure additives, AgrimestMix^®^ and Penergetic-g^®^. The study followed published BMP guidelines [18]. Dairy manure was incubated at two temperatures, representing the temperature in manure storage tanks (20 °C), and the temperature in mesophilic biogas plants (37 °C) (Figure 2). The bioreactors were glass incubation bottles with a 600 mL volume capped with a high-pressure rubber stopper (Bellco Glass Inc., Vineland, NJ, USA). Each bottle was filled with 125 mL of the dairy manure substrate from the Ontario farm and 125 mL of inoculum, for a total of 250 mL of organic material. Additives were then added to the respective bottles, specifically: 7.5 mL (30.3 mL/L) of AgrimestMix^®^, or 105 mg (420 mg/L) of Penergetic-g^®^. After all materials were in the bottles, their weights were recorded and the bottles were capped and sealed using high vacuum grease sealant (Dow Corning, Midland, MI, USA), flushed for 2 min with nitrogen (N_2_), and placed in one of two incubators at the respective temperature.

The inoculum was digestate from a dairy-farm mesophilic biodigester that was degassed and stored for 10 days at 37 °C prior to the start of the study. Three bottles were filled with 125 mL of the inoculum and 125 mL of water to determine the amount of CH_4_ produced by the inoculum. To determine the net CH_4_ produced by the manure substrate, the cumulative CH_4_ produced from the inoculum + water was subtracted from bottles containing dairy manure + inoculum. The trials were stopped when daily gas production fell below 1% of the total cumulative gas produced.

#### 2.3.3. Chemical and Physical Characterization

To standardize CH_4_ production, total solids (TS) and volatile solids (VS) were measured at the beginning of both studies. This was done by drying at 110 °C for 24 h, and loss on ignition at 500 °C, following American Public Health Association (APHA) Standard Methods 2540 B and E. In study #2, the composition of the mixture in each bottle was characterized twice, once at the study set-up and again at the end of the incubation to quantify changes in manure composition. These samples were analyzed for TS, VS, pH and nitrogen at SGS Agri-food Laboratories following recommended methods of analysis [19]. Total nitrogen (TN) was determined using the Dumas method of combustion, total ammoniacal-N (TAN) was measured with an ion-selective electrode, and pH by ion selective electrode. Potential treatment effects on the concentration of each analyte were evaluated using a one-way analysis of variance (ANOVA) in Sigmaplot 13 (Systat Software Inc., San Jose, CA, USA).

#### 2.3.4. Gas Measurements

The volume of biogas in the headspace was measured and the concentration of CH_4_ in the biogas was analyzed by gas chromatography in both studies using a micro gas chromatograph. Certified standard gases were regularly analyzed to ensure accuracy of the Micro-GC.

**Figure 3:**
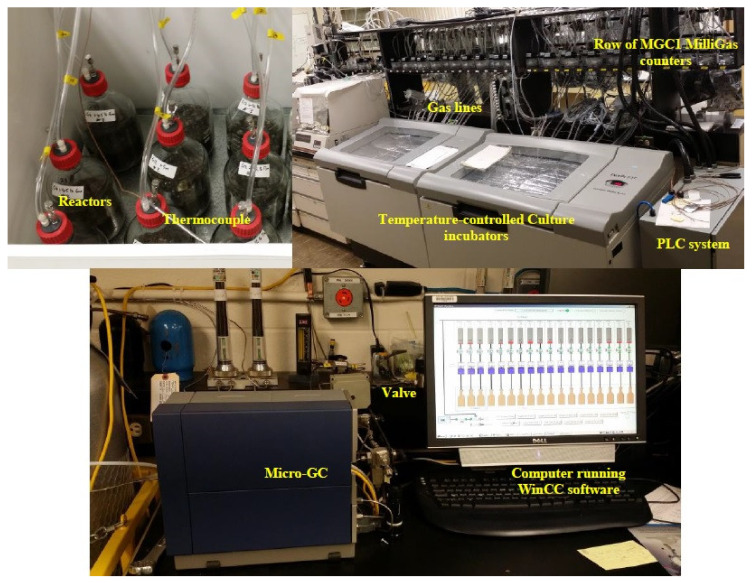
InnoTech Alberta’s BMP batch culture automated sampling system including the programmable logic controller (PLC). The biogas produced was measured using a Milligas counter and gas composition analyzed by a CP4900 Micro-GC.

For study #1, sampling was automated with the volume of biogas produced measured in real-time using a Milligas counter type MGC-1 (Ritter GmbH, Bochum, Germany) via a computer-controlled experimental set-up (Figure 3). The automated process included engaging the sample valve function for sampling and venting of the biogas storage bag, triggering of an CP4900 Micro-GC (Agilent, Santa Clara, CA, USA) for biogas composition analysis and data normalization, integration and logging in real-time via a WinCC human–machine interface software (Siemens, Munich, Germany).

For study #2, sampling was done manually with pressure in the bottle headspace measured using a digital pressure sensor (VWR traceable pressure gauge, Radnor, PA, USA) equipped with a 21-gauge needle to penetrate the rubber septum. A 10 mL headspace sample was taken using a 21-gauge needle and a glass syringe (Agilent, Santa Clara, CA, USA) and injected into the Micro-GC (490 Micro GC, Agilent, Santa Clara, CA, USA) equipped with Molsieve 5A and PoraPLOT Q columns. The CH_4_, CO_2_, O_2_, and N_2_ gas concentrations in the sample were measured. After sampling, the bottles were vented using a syringe to release the remaining headspace pressure and returned to the incubators.

#### 2.3.5. Calculations

For study #1, the volume of biogas was continuously measured by the Milligas counter. For study #2, the volume of biogas produced over the sampling period, V_produced_ (in mL), was calculated using the rearranged perfect gas equation:

V_produced_ = (P_1_ * V_1_ * T_2_/T_1_ * P_2_) − V_1_ (1)

where

P_1_ = pressure measured inside the bottle at the end of the sampling period (kPa)

V_1_ = headspace volume (mL; calculated by subtracting the volume of substrate and inoculum from the total bottle volume)

T_1_ = incubation temperature (K)

P_2_ = ambient pressure (kPa)

T_2_ = ambient room temperature (K)

For both studies, the volume of CH_4_ produced by each bioreactor was calculated using the produced biogas volume, V_produced_ (mL), and the measured concentration of CH_4_ in the biogas on each sampling date, CH_4 conc_ (%),

CH_4 produced_ = V_produced_ * (CH_4 conc_/100) (2)

At the end of each trial, after summing the total CH_4_ produced in each bottle, the portion of CH_4_ production attributed to the dairy manure substrate was determined by subtracting the amount produced by the inoculum + water treatment.

CH_4 manure_ = ΣCH_4 produced_ − ΣCH_4 inoculum_ (3)

Finally, the CH_4_ yield, CH_4 yield_ (mL CH_4_/g VS) was calculated by standardizing the volume of CH_4_ produced by the initial mass of volatile solids from dairy manure in each bottle,

CH_4 yield_ = CH_4 manure_/VS (4)

where

VS = mass of volatile solids from manure at the beginning of the study (g).

There were four trials in total (study #1: cellulose, manure; study #2: 20 °C, 37 °C). Each trial was stopped when gas production slowed to less than 1% per day. Within each trial, significant differences between the treatments were tested using a one-way ANOVA to compare cumulative CH_4_ yields between additives and controls.

## 3. Results

### 3.1. Study #1

#### 3.1.1. Substrate Characterization

Before the BMP test, the cellulose substrate had a TS of 99.5 ± 0.02% and VS of 99.4 ± 0.03%. The dairy manure TS was 8.4 ± 0.06% and VS was 7.0 ± 0.01%.

#### 3.1.2. Methane Emissions

**Figure 4:**
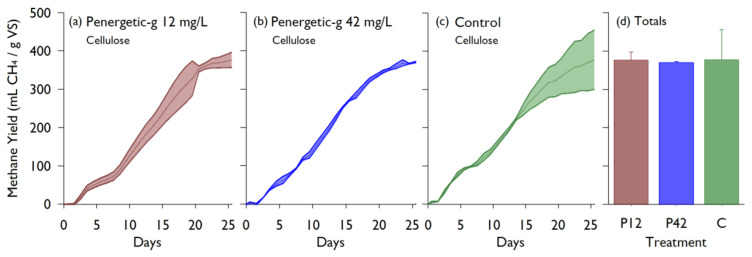
Cumulative methane yields over time from cellulose BMP batch cultures containing the Penergetic-g^®^ additive at 12 mg/L (**a**), 42 mg/L (**b**), or without additive (**c**). Each timeseries shows the mean ± standard deviation (n = 3). The total yield for all treatments are compared in the bar graph, with the error bars showing standard deviation (**d**). Yields are scaled by volatile solids (VS).

The cellulose BMP test compared two rates of the Penergetic-g^®^ additive to a control treatment for 26 days. All cellulose BMP tests resulted in CH_4_ yields within the acceptable range for cellulose (352 to 414 mL/g VS [15]), and there were no significant differences in CH_4_ production between treatments. The amount of CH_4_ produced was 377.2 ± 20.0 mL/g, 370 ± 1.9 mL/g, and 378 ± 77.7 mL/g VS for the low and high rates of Penergetic-g^®^, and the control, respectively (Figure 4).

**Figure 5:**
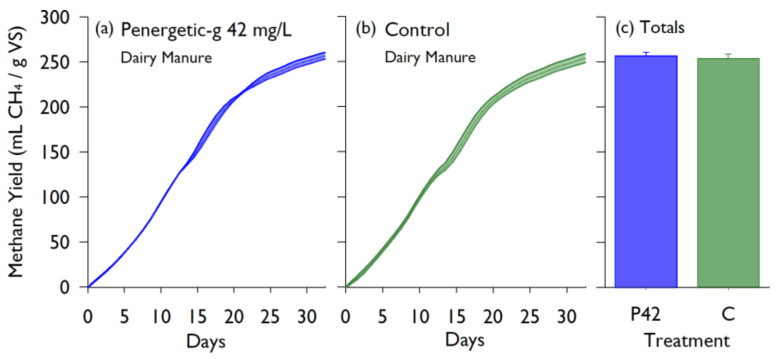
Cumulative methane yields over time from dairy manure BMP batch cultures containing the Penergetic-g^®^ (P) additive at 42 mg/L (**a**), or without additive (**b**). Each timeseries shows the mean ± standard deviation (n = 3). The total yield for all treatments are compared in the bar graph, with the error bars showing standard deviation (**c**). Yields are scaled by volatile solids (VS).

Similarly, no significant difference in CH_4_ production was found for dairy manure with 42 mg/L Penergetic-g^®^ incubated for 32 days at 38 °C. The Penergetic-g^®^ treatment produced an average of 256.9 ± 3.7 mL CH_4_/g VS compared to the control which produced 253.9 ± 5.1 mL CH_4_/g VS (Figure 5).

### 3.2. Study #2

#### 3.2.1. Substrate Characterization

In the second experiment, dairy manure had TS of 9.2 ± 0.12% and VS of 7.9 ± 0.11%. The mixture of manure and inoculum prior to adding the additives had an average TS and VS of 6.4 ± 1.3% and 5.3 ± 1.2%, respectively. Adding the additives did not change the TS or VS (*p* > 0.05), but Penergetic-g^®^ did increase the pH slightly (from 7.37 ± 0.03 to 7.45 ± 0.02; *p* < 0.05). Adding AgrimestMix^®^ did not affect pH, which was unchanged at 7.37 ± 0.02.

#### 3.2.2. Methane Emissions

**Figure 6:**
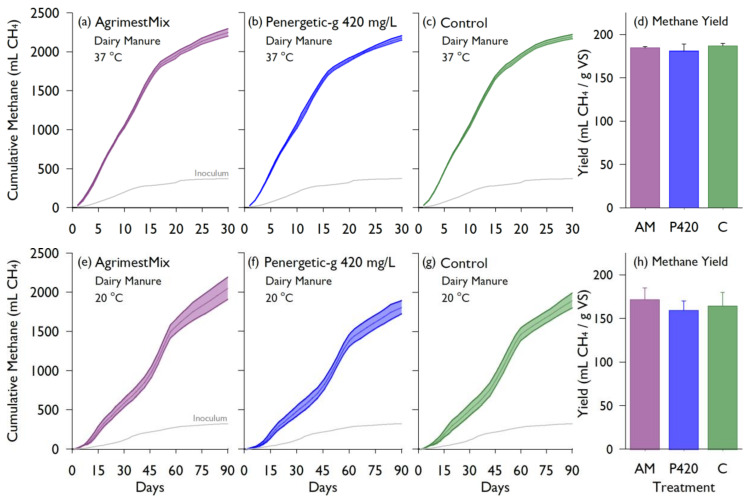
Cumulative methane produced (mL CH_4_) from BMP batch cultures containing liquid dairy manure over a 30-day incubation at 37 °C (**a**–**c**) and a 90-day incubation at 20 °C (**e**–**g**). The bottles contained AgrimestMix (AM) (**a**,**e**), Penergetic-g^®^ at 420 mg/L (**b**,**f**), or a control without additive (**c**,**g**). Each timeseries shows the mean ± standard deviation (n = 3), and the grey line is the average production from inoculum. The total methane yields for each treatment are compared in the bar graph, with error bars showing the standard deviation (**d**,**h**).

The results of the second study were found to be consistent with the first. Regardless of incubation temperature, CH_4_ production was similar among treatments over time (Figure 6, Table 1). After 30 days at 37 °C, the control treatment had a CH_4_ yield of 187 mL/g VS, and there was no significant treatment effect of the additives (*p* = 0.41). Likewise, after 90 days at 20 °C, the control treatment CH_4_ yield was 164.4 mL CH_4_/g VS with no significant treatment effect (*p* = 0.28).

#### 3.2.3. Post-Incubation Substrate Characterization

After the BMP trial was complete, the bottles were opened and sampled to quantify changes in pH, TS, and VS (Table 2). After the 37 °C incubation, all treatments had increased pH and TAN, decreased TS and VS, and stable TN compared to the samples taken before incubation. There was, however, no significant difference among treatments for pH, VS or TAN concentrations. AgrimestMix^®^ had significantly lower TS than the other treatments. The control had significantly lower TN compared to the additive treatments.

After the 20 °C incubation, the pH values were similar to those before incubation. The TS and VS both declined, but not by as much as the 37 °C incubation. Comparing treatments at 20 °C, there were no significant differences for pH, TS, or VS.

## 4. Discussion

Biogas and CH_4_ production by the control treatments at mesophilic temperatures were similar between lab studies. The dairy manure control in lab study #1 produced 348 ± 4 mL/g of VS of biogas with 242 ± 2 mL CH_4_/g VS. In lab study #2, the control treatments produced an average of 330 ± 6 mL/g VS of biogas with 187.0 ± 2.8 mL CH_4_/g VS. These results are similar to values reported in the literature, where dairy manure produced an average of 295 ± 18 mL/g VS of biogas with 204 ± 12 mL CH_4_/g VS of CH_4_ [14], and typical ultimate methane potential (B_0_) used by the Intergovernmental Panel on Climate Change (IPCC) for high producing dairy systems is 240 mL/g VS [20].

Lab study #1 quantified the effects of the Penergetic-g^®^ additive on two types of substrate: microcrystalline cellulose and liquid dairy manure. With cellulose substrate, no significant effect on CH_4_ yield was observed with a low dose of Penergetic-g^®^ (12 mg/L) or the increased rate of 42 mg/L (*p* = 0.964). The source of inoculum could have had an effect on acclimation in some instances, however, manure-based inoculum handles most materials well. The BMP curves in Figure 4 and Figure 5 support that there was a normal progression of methane production during the incubation, indicating that acclimation to cellulose substrate was not an issue. Similarly, no effect was seen when the product was added to liquid dairy manure at 42 mg/L, with no significant effect on the CH_4_ yield (*p* = 0.663). Comparatively, additives used as accelerants such as urea, bentonite, active carbon, and plant ash had much higher total biogas yields (between 485.7–681.9 mL/g VS [21]). In a study comparing a control digester to one using slag as an additive, the control had a methane production of 187.0 ± 2.8 mL CH_4_/g VS while the slag production varied from 397.64–507.29 mL CH_4_/g VS [12]. The methane production from the controls in the above studies are very similar to the production measured in both the Alberta and Ottawa lab studies, however, the various additives all had neutral or increasing effects on the total methane production.

Lab study #2 quantified the effects of AgrimestMix^®^ at the recommended rate for an initial dose, and Penergetic-g^®^ at more than 10 times higher than the recommended rate. Two incubation temperatures were selected to represent mesophilic bio-digestion (37 °C), and summer-time manure storage conditions (20 °C; e.g., Baldé et al. [15]). Bottles incubated at 37 °C produced gas about three times faster than the bottles at 20 °C, but both temperatures produced similar cumulative CH_4_ yields among the treatments. At both temperatures, the CH_4_ yields of additive treatments were not significantly different than the control (*p* > 0.05). Furthermore, the temporal pattern of CH_4_ production was similar for all treatments, with no visual evidence of lag or suppressed methanogenesis over our study period. A similar study evaluating emissions when using Fe salts and composite additives supported that the digestion period can be well represented by a period that covers 80% of the cumulative biogas yield [22].

Comparisons of manure pH, solids, and nitrogen before and after incubation indicated that the additives had small and inconsistent effects on manure composition. This is consistent with the results of Van der Stelt et al. [14], who found that the addition of Agrimest did not change dairy manure characteristics (pH, DM, TN, mineral-N, C:N). The lack of effect on manure composition is also consistent with the lack of effect on CH_4_ production. In contrast, additives such as sulfuric acid reduce the pH to a level that disrupts methanogenesis [6], whereas we observed no treatment effect on pH. Likewise, if biogas production were altered, it would result in a similar change in VS degradation as seen in studies evaluating bio-based carbon, slag, and vermiculite additives [9,23,24]. We observed no treatment effect on VS, which agrees with the CH_4_ results.

## 5. Conclusions

Overall, neither additive affected CH_4_ production under anaerobic conditions in the laboratory. The lack of effect was observed at two temperatures and a range of high doses of additive. If the results from these laboratory incubations are transferrable to farm-scale manure storages, it implies that farms using these additives have normal methane emissions from stored manure. Thus, on-farm methane emission measurements at farms using these additives are expected to be representative of emissions at farms that do not use additives.

## Figures and Tables

**Table 1:** Cumulative net methane production from manure in study #2. Within each incubation temperature, methane yields followed by the same superscript letter are not significantly different.

Treatment	Additive Rate	Temperature	Cumulative CH_4_	CH_4_ Yield
	(mg/L)	(°C)	(mL)	(mL CH_4_/g VS)
AgrimestMix	30	20	1718 ± 142	171.9 ± 13.04 ^a^
Penergetic-g^®^	420	20	1533 ± 145	159.6 ± 10.2 ^a^
Control	None	20	1576 ± 99	164.4 ± 15.4 ^a^
AgrimestMix	30	37	1872 ± 43	184.7 ± 1.4 ^a^
Penergetic-g^®^	420	37	1817 ± 49	181.1 ± 8.0 ^a^
Control	None	37	1856 ± 30	187.0 ± 2.8 ^a^

**Table 2:** Substrate characteristics, pH, total solids (TS), volatile solids (VS), total ammoniacal-N (TAN), and total N (TN) for the control, AgrimestMix^®^, Penergetic-g^®^, and inoculum treatment before and after the BMP test. Within each group (before incubation, after incubation at 37 °C, after incubation at 20 °C), values followed by the same superscript letter are not significantly different.

Treatment	Temp (°C)	pH	TS (%)	VS (%)	TAN (ppm)	TN (%)
*Before Incubation:*						
Control	N/A	7.37 ± 0.03 ^a^	6.41 ± 1.27 ^a^	5.25 ± 1.25 ^a^	1180 ± 105 ^a^	0.383 ± 0.07 ^a^
AgrimestMix^®^	N/A	7.37 ± 0.02 ^a^	6.77 ± 0.05 ^a^	4.64 ± 0.05 ^a^	1213 ± 139 ^a^	0.273 ± 0.06 ^a^
Penergetic-g^®^	N/A	7.45 ± 0.02 ^b^	6.15 ± 0.63 ^a^	4.99 ± 0.63 ^a^	1227 ± 45 ^a^	0.320 ± 0.02 ^a^
*After Incubation:*						
Control	37	7.63 ± 0.01 ^a^	4.07 ± 0.11 ^b^	2.93 ± 0.11 ^a^	1347 ± 57 ^a^	0.190 ± 0.02 ^a^
AgrimestMix^®^	37	7.62 ± 0.02 ^a^	3.73 ± 0.10 ^a^	2.65 ± 0.09 ^a^	1307 ± 12 ^a^	0.283 ± 0.02 ^b^
Penergetic-g^®^	37	7.66 ± 0.07 ^a^	4.08 ± 0.15 ^b^	2.92 ± 0.14 ^a^	1400 ± 279 ^a^	0.277 ± 0.03 ^b^
Control	20	7.39 ± 0.01 ^a^	5.09 ± 0.77 ^a^	3.95 ± 0.76 ^a^	-	-
AgrimestMix^®^	20	7.38 ± 0.01 ^a^	4.51 ± 0.09 ^a^	3.43 ± 0.09 ^a^	-	-
Penergetic-g^®^	20	7.44 ± 0.04 ^a^	4.79 ± 0.31 ^a^	3.64 ± 0.29 ^a^	-	-
